# Supplementary material for: BURRITO: An Interactive Multi-Omic Tool for Visualizing Taxa–Function Relationships in Microbiome Data
Source: Front Microbiol. 2018 Mar 1;9:365. doi: 10.3389/fmicb.2018.00365 (PMC5837987; doi:10.3389/fmicb.2018.00365)
Supplement: Supplementary file 1 [file Data_Sheet_1.DOCX]

## BURRITO: An interactive multi-omic tool for visualizing taxa-function relationships in microbiome data

Colin P McNally^1,†^, Alexander Eng^1,†^, Cecilia Noecker^1,†^, William C Gagne-Maynard^2^, and Elhanan Borenstein^1,3,4,^*

## Supplementary Text

### Separating server-side data processing from browser-side visualization management

As the user selects input files for visualization, these files are uploaded to an R Shiny server for processing. Briefly, the server calculates and formats attributions of function abundance to taxa (when necessary) and average abundances, summarizes abundance and function share to the selected minimum summary level, and orders samples based on group labels (if provided). As the individual components are processed, they are sent to the browser to support visualization.

To enable responsive interaction, all user-triggered real-time summarizing and expansion of hierarchy levels is handled by the browser. To achieve this, the browser maintains information for all currently displayed taxonomic abundances and function abundance share attributions for bar highlighting and tooltip information in addition to information for all taxonomic abundances and function abundance share attributions at the minimum requested hierarchy levels. The visualization is thus able to expand or collapse nodes in either hierarchy and update the abundance plots as necessary without communicating with the server.

### Ordering and visually grouping samples by user-provided labels

Users can control the organization of samples in both abundance plots by uploading a table mapping sample IDs to sample labels. Once a table is chosen and the user selects the column of the table to arrange by, the visualization places samples adjacent to samples with the same group label. This grouping is shown in the visualization by a colored background behind the sample IDs within that group, with the label for the group displayed below. The groups will appear in the order of first appearance of group label in the grouping table, such that if a sample is mapped to label “A” in a row before any row that maps a sample to label “B”, then group “A” will appear to the left of group “B” in the visualization. Additionally, samples will be similarly ordered within the group by the order they appear in the grouping table.

### Distributing abundances of hierarchy entities that belong to multiple entities in a higher hierarchy level

BURRITO allows users to upload custom taxonomic and functional hierarchies with many-to-one mappings from higher level entities to lower level entities. In fact, the default functional hierarchy used by BURRITO has such many-to-one mappings, and will serve here as an example of how BURRITO processes such hierarchies. In the default functional hierarchy, KOs can belong to multiple subpathways, superpathways, or categories. When summarizing KO abundances to a higher functional hierarchy level, BURRITO evenly distributes the abundance of the KO across all entities in that hierarchy level that the KO belongs to. Similarly, the share of that KO attributed to a given taxon is also distributed evenly among those higher level entities. If a custom taxonomic or functional hierarchy is uploaded with similar many-to-one mappings, the same even distribution of abundances and shares is performed when summarizing to higher hierarchy levels.

### Assigning colors to taxa and functions based on hierarchical relationships

The default color scheme for the visualization uses the ColorBrewer categorical color scales as a starting point (Brewer, 2017), assigning a “home color” for each phylum from the palette “Set3” and for each functional category from the palette “Set1”. If there are more phyla or functional categories than colors available in the main palettes, colors from the palette “Dark2” are added as needed. Multiple taxonomic kingdoms are assigned shades of gray. Shades of the core colors are generated for the taxonomic and functional subcomponents by dividing up a surrounding color space defined by the HCL (hue, chroma, lightness) color scheme. The shades vary in terms of all three features but remain identifiable as originating from the core phylum/category color. The separation between colors for individual nodes decreases as the number of nodes increases.

Alternatively, a user can choose to have a random categorical color scheme for each taxonomic and functional node (regardless of hierarchy level), which uses assorted colors from multiple ColorBrewer palettes.
